# AI-Driven Advancements in Orthodontics for Precision and Patient Outcomes

**DOI:** 10.3390/dj13050198

**Published:** 2025-04-30

**Authors:** David B. Olawade, Navami Leena, Eghosasere Egbon, Jeniya Rai, Aysha P. E. K. Mohammed, Bankole I. Oladapo, Stergios Boussios

**Affiliations:** 1 Department of Allied and Public Health, School of Health, Sport and Bioscience, University of East London, London E16 2RD, UK; 2 Department of Research and Innovation, Medway NHS Foundation Trust, Gillingham ME7 5NY, UK; stergios.boussios@kmms.ac.uk; 3 Department of Public Health, York St John University, London E14 2BA, UK; jeniya.rai@yorksj.ac.uk; 4 School of Health and Care Management, Arden University, Arden House, Middlemarch Park, Coventry CV3 4FJ, UK; aparveen@arden.ac.uk; 5 Faculty of Public Health and Healthcare Management, Westford University College, Sharjah 50325, United Arab Emirates; drnavami@gmail.com; 6 Department of Tissue Engineering and Regenerative Medicine, Faculty of Life Science Engineering, FH Technikum, 1200 Vienna, Austria; eghosaseregabriel@gmail.com; 7 School of Science and Engineering, University of Dundee, Dundee DD1 4HN, UK; bioladapo@abuad.edu.ng; 8 Faculty of Medicine, Health, and Social Care, Canterbury Christ Church University, Canterbury CT1 1QU, UK; 9 Faculty of Life Sciences & Medicine, School of Cancer & Pharmaceutical Sciences, King’s College London, London WC2R 2LS, UK; 10 Kent Medway Medical School, University of Kent, Canterbury CT2 7NZ, UK; 11 Department of Medical Oncology, Medway NHS Foundation Trust, Gillingham ME7 5NY, UK; 12 AELIA Organization, Thermi, 57001 Thessaloniki, Greece

**Keywords:** artificial intelligence, personalized orthodontics, tooth movement prediction, aligner fabrication, remote monitoring, dental care

## Abstract

Artificial Intelligence (AI) is rapidly transforming orthodontic care by providing personalized treatment plans that enhance precision and efficiency. This narrative review explores the current applications of AI in orthodontics, particularly its role in predicting tooth movement, fabricating custom aligners, optimizing treatment times, and offering real-time patient monitoring. AI’s ability to analyze large datasets of dental records, X-rays, and 3D scans allows for highly individualized treatment plans, improving both clinical outcomes and patient satisfaction. AI-driven aligners and braces are designed to apply optimal forces to teeth, reducing treatment time and discomfort. Additionally, AI-powered remote monitoring tools enable patients to check their progress from home, decreasing the need for in-person visits and making orthodontic care more accessible. The review also highlights future prospects, such as the integration of AI with robotics for performing orthodontic procedures, predictive orthodontics for early intervention, and the use of 3D printing technologies to fabricate orthodontic devices in real-time. While AI offers tremendous potential, challenges remain in areas such as data privacy, algorithmic bias, and the cost of adopting AI technologies. However, as AI continues to evolve, its capacity to revolutionize orthodontic care will likely lead to more streamlined, patient-centered, and effective treatments. This review underscores the transformative role of AI in modern orthodontics and its promising future in advancing dental care.

## 1. Introduction

Orthodontics, a specialized branch of dentistry, plays a crucial role in diagnosing, preventing, and correcting irregularities in the alignment of teeth and jaws [1,2]. These irregularities, often referred to as malocclusions, not only affect aesthetics but also impact oral functionality, including chewing, speaking, and maintaining proper oral hygiene [3]. Traditionally, orthodontic treatment planning has been a labor-intensive process, reliant on the orthodontist’s expertise in manually evaluating a patient’s dental structure, assessing X-rays, and making subjective predictions about tooth movement over time. This manual approach, while effective, is subject to variability in outcomes, treatment duration, and patient experience [4].

Historically, orthodontic treatment options such as braces and aligners have been standardized, with fixed regimens applied across a broad range of patients. The challenge with such an approach lies in the fact that each individual’s dental structure and biology are unique, leading to varied responses to treatment. The one-size-fits-all approach can result in slower progress for some patients, extended treatment times, and, in some cases, suboptimal results [5,6]. Moreover, the complexity of predicting how a patient’s teeth will move under mechanical forces like braces and aligners adds an additional layer of uncertainty to traditional orthodontic care [7].

The introduction of Artificial Intelligence (AI) into orthodontics is transforming this field by enabling a more precise, personalized approach to treatment planning. AI technologies, particularly those driven by machine learning (ML) and computer vision, have the capacity to analyze patient-specific dental data—such as 3D scans, X-rays, and intraoral photos—at a granular level that exceeds human capability [8]. By leveraging vast datasets from previous orthodontic cases and outcomes, AI algorithms are trained to predict tooth movement more accurately, identify optimal forces to apply, and create tailored treatment plans for individual patients [9].

This shift toward personalized orthodontic care powered by AI is a game-changer. AI-driven systems can optimize the design of orthodontic devices (like braces and aligners) for each patient, factoring in the unique characteristics of their teeth and jaw alignment. As a result, patients can experience faster, more efficient treatments with fewer complications or adjustments [10,11]. The predictive capabilities of AI also offer the potential to forecast how long the treatment will take, making the process more transparent and reducing the uncertainty that patients often face during orthodontic care. Furthermore, AI enables continuous monitoring of a patient’s progress throughout the treatment journey [12,13]. Using advanced image recognition and predictive modeling, AI systems can assess whether tooth movement is proceeding as expected and provide real-time recommendations to adjust the treatment plan if needed. This level of precision and adaptability is difficult to achieve through traditional methods, making AI-powered solutions an exciting development in orthodontics [14].

The rationale for exploring the application of AI in personalized orthodontic treatment planning lies in the need to enhance precision and efficiency in dental care. Traditional orthodontic methods, while effective, often rely on subjective evaluations and standard treatment protocols that do not account for individual patient variability. This results in longer treatment durations, unpredictable outcomes, and frequent adjustments, which can be both costly and inconvenient for patients [15]. The problem addressed in this review is the limited ability of traditional methods to optimize treatment for each patient, leading to inefficiencies and potential patient dissatisfaction. AI has the potential to revolutionize orthodontic care by providing more accurate predictions of tooth movement, tailoring treatment plans based on patient-specific data, and continuously adjusting the treatment process in real time. The objective of this review is to critically assess how AI can be integrated into orthodontic practice to overcome these limitations, improving both the effectiveness and efficiency of treatment, and offering a roadmap for future advancements in AI-driven orthodontic care.

## 2. AI in Orthodontic Treatment

AI is revolutionizing the orthodontic field, offering precision and efficiency that were previously unattainable with traditional methods. By harnessing ML, neural networks, and computer vision, AI enhances the orthodontist’s ability to create personalized, data-driven treatment plans [7,16]. These advanced AI systems analyze vast amounts of patient-specific dental data, including 3D scans, X-rays, and detailed models of teeth and jaw structures, to predict how teeth will respond to different treatments [17]. This allows orthodontists to customize aligners or braces for each patient, resulting in faster, more effective treatments with fewer complications or delays [18].

### 2.1. Data Collection: Creating a Detailed Model

**Figure 1:**
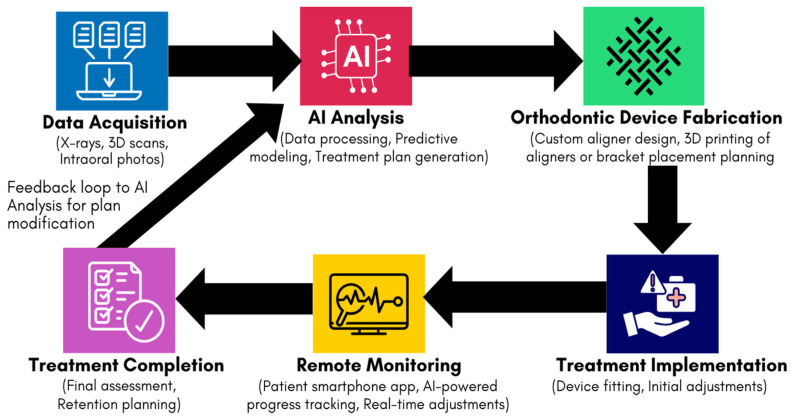
Schematic diagram of the AI-assisted orthodontic workflow, demonstrating the integration of data acquisition, AI analysis, device fabrication, treatment implementation, remote monitoring, and treatment completion.

At the core of AI-powered orthodontic treatment is data collection, where patient-specific information is gathered to create a detailed digital model of the dental anatomy. AI systems utilize high-resolution 3D scans, intraoral photographs, and X-rays to capture a complete view of the patient’s teeth and jaw structure [17]. These datasets provide the foundation upon which AI algorithms can work, offering detailed insights into the position, alignment, and spacing of the teeth [19]. With advancements in imaging technologies, particularly the rise of 3D scanning, the data that orthodontists can now collect is far more detailed and accurate than in the past [20]. AI-driven imaging devices can even map the underlying bone structures and surrounding tissues, allowing for a comprehensive understanding of the patient’s unique oral characteristics [21]. This level of detail helps ensure that the AI system’s predictions and recommendations are as precise as possible, tailored to the patient’s exact dental anatomy. Figure 1 illustrates the typical workflow of AI-assisted orthodontics, from initial data acquisition to treatment completion.

### 2.2. AI-Based Prediction: Understanding Tooth Movement

Once the data have been collected, AI algorithms are used to predict how a patient’s teeth will move in response to different treatment options. ML models are trained on vast amounts of historical orthodontic data, including thousands of previous cases with different types of malocclusions and treatments. These models, often powered by deep learning neural networks, can analyze how teeth shift over time based on the forces applied by braces, aligners, or other orthodontic devices [22]. AI excels in understanding the biomechanical principles that govern tooth movement, including how pressure and force need to be applied to achieve the desired positioning. For instance, AI models can predict the rate at which teeth will move, which teeth need additional force, and how specific teeth may respond to orthodontic appliances [12,23]. This predictive capability significantly enhances the orthodontist’s ability to plan treatments more effectively, allowing for adjustments that maximize efficiency and reduce treatment time [18]. Current trends in AI-driven orthodontic predictions also include the use of dynamic simulations. These simulations enable orthodontists to visualize how a patient’s teeth will move over the course of the treatment. By running multiple simulations with varying treatment options, AI systems can recommend the most effective approach for each patient, minimizing trial-and-error methods and leading to faster, more accurate outcomes [24].

### 2.3. Personalized Treatment Planning: Tailored to the Patient

AI-powered orthodontics takes personalization to a new level by customizing treatment plans for each individual patient based on their unique dental structure and predicted tooth movement. Rather than relying on generalized protocols, AI-driven systems analyze patient-specific data and create an optimized treatment plan that details the exact design, placement, and force application of orthodontic appliances such as braces or clear aligners [9,25]. Personalized treatment planning with AI includes the design of custom-made aligners that are optimized for each stage of the patient’s tooth movement. In clear aligner therapy (e.g., Invisalign), AI systems generate a sequence of aligners that apply precise amounts of force at each stage of treatment, moving the teeth gradually into their ideal positions [26]. The aligners are designed to ensure that the teeth move in the shortest possible time while minimizing discomfort for the patient.

For traditional braces, AI helps orthodontists determine the best placement of brackets and wires, ensuring that the right amount of pressure is applied to the teeth. AI also assists in determining the frequency of adjustments and the need for auxiliary tools like rubber bands or headgear, making the treatment plan more efficient and patient-friendly [10]. The personalization of orthodontic care through AI does not stop at the beginning of treatment. AI systems are dynamic and adaptable, continuously refining the treatment plan as the patient’s teeth move. This ability to adjust treatment in real-time allows for more precise corrections, reducing the need for extensive follow-up visits or mid-treatment adjustments [27].

### 2.4. Continuous Monitoring and Adjustment: Real-Time Progress Tracking

One of the most significant advantages of AI in orthodontics is its ability to provide continuous monitoring of the patient’s progress throughout the treatment process. AI systems can track how the teeth are moving and compare the real-time data against the predicted movement patterns [28]. This allows orthodontists to ensure that the treatment is proceeding as planned and to make any necessary adjustments before problems arise [10,29]. Patients can also play an active role in this monitoring process. With the rise of tele-orthodontics and the use of smartphone apps, patients can submit photos of their teeth from home. AI-driven systems analyze these images and generate reports on the patient’s progress, alerting both the patient and the orthodontist if there are any deviations from the treatment plan [30]. This type of remote monitoring is particularly valuable for patients with clear aligners, who may not need frequent in-office visits but still require regular check-ins to ensure their treatment stays on track [31]. Continuous monitoring powered by AI enables orthodontists to make timely interventions when necessary. For example, if a tooth is not moving as expected, the AI system may suggest an adjustment to the aligner design or bracket placement. This ensures that treatment remains on course, preventing delays or the need for more extensive corrections later. In some cases, AI systems can even predict when a patient’s treatment is nearing completion and suggest early removal of braces or aligners, further enhancing the overall efficiency of the process.

## 3. Applications in Orthodontic Care

The use of AI in orthodontics has expanded significantly in recent years, providing powerful tools to enhance personalized treatment planning. Through advanced algorithms and ML models, AI offers more accurate predictions of tooth movement, customizes aligners with precision, optimizes treatment time, enables real-time monitoring, and improves overall treatment planning with 3D visualization. These applications not only improve the efficiency of orthodontic care but also enhance patient outcomes [32,33,34].

Several applications of AI in orthodontic care have demonstrated significant advancements in accuracy, treatment duration, patient satisfaction, appointment efficiency, and predictive performance. AI-enhanced diagnostic tools have shown higher accuracy in treatment planning, with studies reporting significantly improved precision compared to traditional approaches (*p* < 0.05) [35]. AI models predicting orthodontic treatment outcomes achieved 73% accuracy, though limitations persist in complex cases [36]. A meta-analysis further found that AI-based orthodontic treatment planning had an overall accuracy of 95.47% [15]. Regarding treatment duration, AI-assisted planning reduced overall treatment time by an average of 4.3 months compared to conventional methods (mean ± SD: 14.6 ± 3.2 months vs. 18.9 ± 4.5 months, *p* < 0.001) [35]. Additionally, machine learning models have provided more precise estimates of orthodontic treatment duration, with a mean absolute error of 7.27 months [4]. Patient satisfaction also increased with AI-driven treatment planning, as evidenced by significantly higher satisfaction scores (mean ± SD: 9.2 ± 0.6 vs. 8.1 ± 0.8, *p* < 0.001) compared to traditional methods (mean ± SD: 9.2 ± 0.6 vs. 8.1 ± 0.8, *p* < 0.001) compared to traditional methods [35]. A separate study found that AI-predicted post-treatment outcomes were better received among laypersons, whereas orthodontists exhibited more critical evaluations [37]. AI also contributed to reducing the number of required orthodontic appointments, with a lower mean number of visits (10.2 ± 2.1 vs. 12.8 ± 3.4, *p* < 0.001) [35]. In terms of predictive performance, AI outperformed conventional linear regression in 6 out of 32 soft-tissue landmarks when predicting orthodontic outcomes [38]. AI models also showed high predictive accuracy for facial changes post-treatment, though their reliability in predicting lip-to-teeth relationships remained lower [39]. These findings highlight AI’s growing role in optimizing orthodontic care, making treatments more accurate, efficient, and patient-centered.

Table 1 classifies different patient groups based on age and malocclusion type, highlighting how AI-powered orthodontics benefits each category. AI’s role varies based on patient needs, ranging from early diagnosis in children to AI-driven aligner fabrication for adults and surgical prediction modeling for severe cases. While AI improves treatment efficiency, precision, and monitoring, considerations such as growth patterns in younger patients, compliance in teenagers, and periodontal health in older adults must be addressed. This classification ensures a more structured and patient-centered perspective on AI’s impact in orthodontic treatment.

### 3.1. Prediction of Tooth Movement

One of the cornerstone applications of AI in orthodontics is its ability to predict tooth movement with a high degree of accuracy. In traditional orthodontic treatment, predicting how teeth will respond to braces or aligners involves subjective expertise and an understanding of biomechanical forces [41]. AI, however, leverages vast amounts of historical treatment data and sophisticated biomechanical models to simulate how each tooth will move over time.

AI algorithms are trained on large datasets of past treatments, which include various malocclusion types, patient-specific dental structures, and orthodontic appliances. This wealth of data enables AI to create highly accurate models of tooth movement, predicting not only the final position of each tooth but also the timeline over which these movements will occur [47]. Research demonstrates that AI-driven biomechanics can simulate the effects of different forces applied to teeth through braces, clear aligners, or other orthodontic devices. This level of precision allows orthodontists to determine the ideal placement of brackets or the design of aligners to achieve the best results in the shortest time possible [48,49].

Recent studies show that AI-based prediction models outperform traditional methods in both accuracy and speed of prediction. For example, a study found that Invisalign’s AI-driven system achieved a mean accuracy of 50% for tooth movements, with specific movements, such as buccal-lingual crown tips, reaching up to 56% accuracy [50]. Another study demonstrated that AI-based predictions could reduce overall treatment time by several months in complex cases, as AI allows for more targeted interventions based on real-time progress [51].

### 3.2. Custom Aligner Fabrication

Clear aligners, such as those used in systems like Invisalign, have become increasingly popular due to their aesthetic appeal and convenience. However, their effectiveness relies heavily on the precision of their design [26]. AI technologies play a critical role in the fabrication of custom aligners that are uniquely tailored to each patient’s dental anatomy. AI algorithms analyze 3D scans of a patient’s teeth and jaw structure to generate aligners that apply the precise amount of force required to move each tooth [17,26,52]. Unlike traditional aligners, which may rely on standard templates, AI-driven aligners are based entirely on the individual’s dental characteristics, ensuring that the treatment is more comfortable and efficient [53]. The aligners are also designed to apply continuous, gentle pressure on the teeth, optimizing the force distribution to ensure smooth, predictable movements [54].

AI not only improves the accuracy of aligner design but also speeds up the production process. Traditional aligner manufacturing can take weeks, requiring manual intervention at several stages. In contrast, AI-driven production systems can automate many aspects of the design and fabrication process, allowing for faster delivery of custom aligners to patients [52]. This acceleration in production time is particularly beneficial for patients undergoing incremental aligner treatment, as they receive their next set of aligners more quickly, keeping their treatment on schedule [31]. A study found that AI-generated aligners resulted in a 20% reduction in the number of aligners needed per treatment case, as fewer mid-course corrections were required [41]. Additionally, patient-reported discomfort was lower due to the more precise fit of the aligners, which conformed more closely to the natural contours of the patient’s teeth and gums.

### 3.3. Optimization of Treatment Time

One of the most significant advantages of AI in orthodontics is its ability to optimize treatment time. Orthodontic treatments can be lengthy, often taking 18 to 24 months or more, depending on the severity of the case [6]. AI shortens this duration by ensuring that each stage of the treatment is as efficient as possible [10,15,42]. AI systems simulate tooth movement and optimize the forces applied by orthodontic appliances to ensure that each tooth moves in the desired direction with minimal delays. The algorithms can suggest modifications to the treatment plan if certain teeth are not moving as predicted or if adjustments are needed to maintain progress [55]. By continuously refining the treatment plan based on real-time data, AI helps avoid unnecessary delays or stagnation in tooth movement, allowing patients to complete their treatment faster than with traditional methods [11,32].

AI can also assess whether a combination of treatment modalities, such as the use of both traditional braces and clear aligners, would yield faster results. For instance, some cases may benefit from starting with braces to correct more severe misalignments, followed by aligners for fine-tuning. AI’s ability to simulate different treatment approaches enables orthodontists to make evidence-based decisions on the most time-efficient strategy for each patient. Clinical evidence supports the time-saving potential of AI in orthodontics [56,57,58]. According to a study, AI has been demonstrated to drastically enhance the efficiency of orthodontic practices, reducing analysis time by as much as 80-fold compared to traditional manual methods [59].

### 3.4. Enhanced Patient Monitoring and Adjustments

AI-powered orthodontic tools offer enhanced patient monitoring, allowing orthodontists to track treatment progress in real time. This level of monitoring is especially beneficial for patients using clear aligners, as it allows orthodontists to ensure that the aligners are working as intended without requiring frequent in-office visits [42,60]. Using AI-driven smartphone applications, patients can submit images of their teeth from home. These images are then analyzed by AI algorithms to assess whether tooth movement is proceeding according to plan [61]. If the AI detects any deviations from the expected movement patterns, it can alert the orthodontist, who can then recommend adjustments or changes to the treatment plan. This remote monitoring capability is particularly valuable for patients who live far from their orthodontist or have busy schedules, as it reduces the need for unnecessary office visits [31,62].

AI’s ability to track progress in real time means that orthodontists can make proactive adjustments to the treatment plan, ensuring that any issues are addressed early on. For example, if a tooth is not moving as expected, the AI system may suggest modifications to the aligner design or bracket positioning, preventing delays and ensuring that the treatment stays on course [41,63]. This level of personalization helps maintain the efficiency of the treatment and reduces the risk of complications [42]. A study by Sosiawan et al. demonstrated that AI-driven remote monitoring systems, such as Dental Monitoring, can reduce the number of in-office visits by a significant margin without compromising treatment outcomes [62]. Patients using AI-assisted monitoring systems reported high satisfaction levels, citing the convenience and accessibility of the technology. Moreover, orthodontists noted that the real-time insights provided by AI allowed for quicker interventions, resulting in more effective treatment outcomes [10,62].

### 3.5. 3D Visualization for Treatment Planning

AI-powered software enables orthodontists to create detailed 3D models of a patient’s teeth and jaw, which are used to plan every aspect of the treatment process [64]. These 3D visualizations provide a comprehensive view of the patient’s current dental structure and simulate how the teeth will move over time [49]. By using AI-generated 3D models, orthodontists can simulate the entire treatment process, from the initial positioning of the teeth to the final outcome. These simulations help orthodontists identify potential challenges early on and adjust the treatment plan to address any issues. The ability to visualize the expected outcome also allows orthodontists to communicate the treatment plan more effectively to patients, helping them understand the process and what to expect [64].

AI-driven 3D visualization tools offer patients a clearer understanding of their treatment journey. Patients can see a step-by-step projection of how their teeth will move over time, which enhances their engagement and motivation. Research shows that when patients have a visual representation of the expected results, they are more likely to adhere to the treatment plan and follow the orthodontist’s recommendations [65]. A study demonstrated that patients who were shown AI-driven 3D treatment visualizations before starting their treatment reported higher levels of satisfaction and adherence [9,64]. Another study also found that patients with access to these visualizations had fewer missed appointments and a greater commitment to wearing their aligners as directed [30].

### 3.6. Accuracy and Reliability of AI-Assisted Tracing Systems in Orthodontics

AI-powered automatic tracing systems have significantly enhanced the efficiency of cephalometric analysis, reducing the time required for landmark identification and measurement. These AI-driven tools leverage deep learning algorithms to detect anatomical landmarks on radiographic images, facilitating more streamlined orthodontic diagnostics and treatment planning [40]. However, the accuracy and reliability of these systems remain a subject of scrutiny, as automated tracing tools may still misidentify landmarks, particularly in complex cases with anatomical variations, overlapping structures, or poor image quality [10].

#### 3.6.1. Comparison of Manual Tracing vs. AI-Assisted Tracing

Manual cephalometric tracing, performed by orthodontists, has long been the gold standard due to the practitioner’s ability to interpret complex anatomical structures, account for variations, and make necessary adjustments based on clinical experience [42]. Although time-consuming, manual tracing remains highly reliable, particularly in cases where image distortion, pathology, or unusual skeletal patterns make automatic detection challenging.

AI-assisted tracing systems, on the other hand, offer speed and automation but are not without limitations. Studies have shown that while AI-based tracing tools can achieve high accuracy rates—often within 1–2 mm deviation from manual tracing—certain landmarks, such as the orbitale, condylion, and gonion, are more prone to misidentification [66]. Research comparing AI and manual tracing has reported error rates ranging from 2% to 10%, depending on the quality of the AI algorithm and the dataset used for training. Some studies suggest that AI can achieve up to 90–95% accuracy for well-defined landmarks but may struggle in cases where structures are partially obscured or where anatomical variance exists [67].

#### 3.6.2. Need for Manual Corrections and Hybrid Approaches

Due to these inherent challenges, AI-assisted tracing systems often require manual corrections by orthodontists to ensure clinical accuracy. Many modern AI-based tools now incorporate hybrid approaches, where the system performs the initial landmark detection, and the orthodontist reviews and refines the tracing as needed [68]. This combination optimizes efficiency while maintaining diagnostic precision, ensuring that errors in landmark identification do not lead to incorrect treatment planning. Furthermore, advancements in adaptive learning algorithms are gradually improving AI accuracy by allowing systems to refine their predictions based on expert feedback [69,70]. These systems can learn from corrections made by orthodontists, thereby improving their performance over time and reducing the margin of error in subsequent analyses.

## 4. Benefits of AI-Powered Personalized Orthodontic Treatment

**Figure 2:**
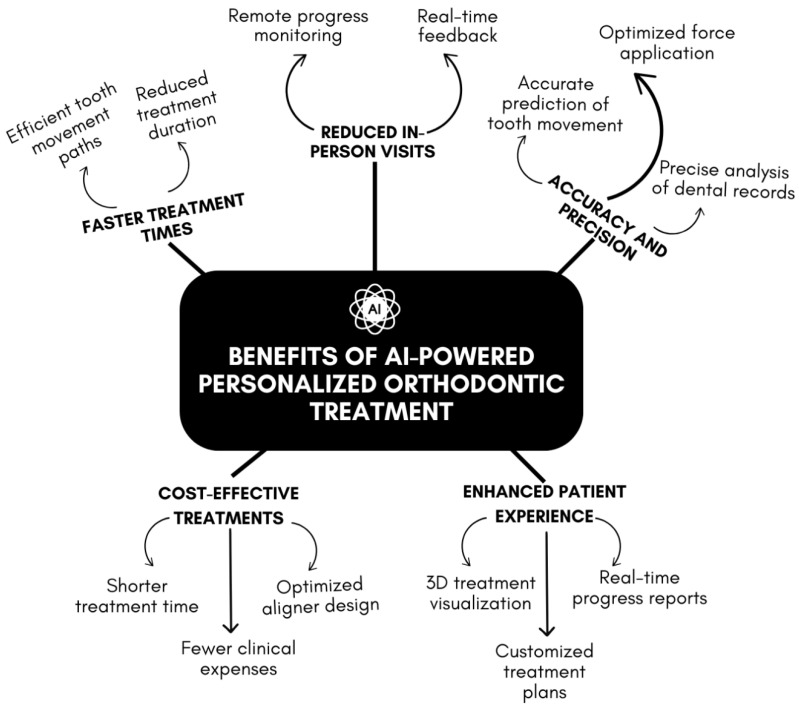
Overview of the benefits of AI-powered personalized orthodontic treatment.

The integration of AI into orthodontic treatment offers numerous benefits that significantly enhance both clinical outcomes and patient experiences. These advantages stem from AI’s ability to deliver highly precise, efficient, and personalized care. Figure 2 illustrates the main benefits of AI-powered personalized orthodontic treatment and their key aspects. AI integration enhances treatment efficiency through faster tooth movement and reduced duration, contributing to faster treatment times. Reduced in-person visits are facilitated by remote progress monitoring and real-time feedback. AI-driven precision is achieved through optimized force application and accurate predictions of tooth movement, ensuring accuracy and precision. These advancements also enable cost-effective treatments by shortening treatment time, optimizing aligner design, and minimizing clinical expenses. The patient experience is further enhanced through 3D treatment visualization and real-time progress reports, enabling customized treatment plans.

Table 2 provides a comprehensive overview of AI applications in orthodontic treatment, distinguishing between the specific machine learning models used and their functional roles in clinical practice. AI has transformed various aspects of orthodontics, from automated cephalometric landmark detection using convolutional neural networks (CNNs) to tooth movement prediction through recurrent neural networks (RNNs). Additionally, custom aligner fabrication benefits from generative adversarial networks (GANs), while automated bracket positioning utilizes object detection algorithms such as Faster R-CNN.

These AI-powered technologies enhance diagnostic accuracy, treatment efficiency, and patient monitoring, reducing human error and improving orthodontic workflow automation. However, limitations remain, including AI’s dependence on high-quality training data, potential errors in complex cases, and the need for orthodontist oversight in AI-generated treatment plans. The integration of AI with 3D printing, robotics, and remote monitoring tools further expands its potential, but challenges such as high implementation costs, patient compliance issues, and variability in AI performance across diverse populations must be addressed.

This structured comparison provides a more technical and detailed perspective on AI’s impact on orthodontic care, emphasizing that AI serves as an assistive tool rather than a replacement for orthodontists. By refining algorithmic models and integrating AI seamlessly into orthodontic workflows, the field can achieve more precise, efficient, and accessible treatments while maintaining the crucial role of human expertise in clinical decision-making.

### 4.1. Accuracy and Precision

One of the primary benefits of AI in orthodontics is its capacity to provide unprecedented levels of accuracy and precision in treatment planning. Traditional orthodontic methods rely heavily on manual evaluations and subjective judgment, which can result in variable outcomes. AI, on the other hand, can analyze large datasets of dental records, 3D scans, X-rays, and treatment outcomes to create precise models of each patient’s dental anatomy [34]. AI’s ability to predict tooth movement more accurately allows for a highly individualized approach to treatment. By simulating different treatment scenarios, AI systems can optimize the force applied to teeth, ensuring that movement is both effective and controlled. This precision results in more predictable outcomes, fewer complications, and a reduced need for mid-treatment adjustments. Clinical evidence suggests that AI-driven predictions of tooth movement can reduce error margins significantly compared to traditional manual assessments, leading to a higher success rate in achieving the desired treatment outcomes [10]. A recent study demonstrated that AI-based orthodontic systems significantly improve the accuracy and precision of treatment planning compared to traditional methods. For instance, a study compared conventional plaster models with AI-assisted virtual 3D models in orthodontics. The results indicated that AI-driven 3D scanning models were not only more accurate but also reduced the time required for measurements by over 60% compared to traditional methods [72].

### 4.2. Faster Treatment Times

AI plays a critical role in reducing the duration of orthodontic treatments by optimizing the movement of teeth. By analyzing patient-specific data and applying sophisticated biomechanical principles, AI systems can determine the most efficient path for tooth movement. This means that each tooth is moved with the appropriate amount of force in the right direction, minimizing unnecessary movements that could prolong treatment times [11,73]. Traditional orthodontic treatments, particularly with braces or aligners, can take 18 to 24 months or longer [74]. However, AI-driven systems can shorten this timeline by identifying the most effective strategies for each patient’s unique dental structure. This reduction in treatment time not only enhances patient satisfaction by providing quicker results but also improves the overall comfort of the treatment process [29]. Research shows that AI-based orthodontic treatments can reduce treatment duration by up to 26%, allowing patients to achieve their desired outcomes faster by minimizing unnecessary movements that could prolong treatment times [73].

### 4.3. Reduced Number of In-Person Visits

One of the major advantages of AI-powered orthodontic treatment is the reduced need for frequent in-person office visits. Traditionally, orthodontic treatments require regular check-ups to assess progress and make any necessary adjustments. With AI-based treatment plans, however, real-time monitoring tools allow patients to track their progress remotely [62]. AI systems can analyze photos or scans submitted by patients via smartphone apps or other devices, providing feedback on whether the treatment is proceeding as expected. If any issues arise or adjustments are needed, the AI system can alert the orthodontist, who can make recommendations or adjustments without requiring the patient to visit the clinic [61]. This remote monitoring capability is particularly beneficial for patients in rural or underserved areas, as it makes orthodontic care more accessible and convenient. As a result, AI-powered orthodontic treatments can significantly reduce the number of in-person visits, saving both time and resources for patients and practitioners [62].

### 4.4. Cost-Effective Treatments

AI-driven personalized orthodontic treatment can also lead to more cost-effective care for both patients and orthodontic practices. One of the primary cost-saving benefits is the reduction in treatment time, which minimizes the number of aligners or braces needed and reduces the frequency of adjustments. By streamlining the treatment process, AI helps lower the overall cost of care. Furthermore, the use of AI in creating custom aligners can reduce manufacturing costs. AI technologies optimize the design and production of aligners by minimizing the number of revisions needed during treatment. This efficiency reduces the cost of materials and labor, making clear aligner treatments more affordable for patients [26,75]. In addition, fewer in-person visits mean fewer clinical expenses, contributing to lower overall treatment costs. As AI continues to evolve, its ability to deliver high-quality, personalized orthodontic care at a reduced cost will make it more accessible to a broader range of patients.

### 4.5. Enhanced Patient Experience

AI has the potential to significantly improve the overall patient experience in orthodontic care. The personalized nature of AI-driven treatments means that patients receive customized aligners or braces tailored specifically to their unique dental structure, ensuring a more comfortable fit and less discomfort during the treatment process. By optimizing tooth movement and reducing treatment time, AI makes orthodontic care more comfortable and efficient, leading to higher patient satisfaction [76]. Additionally, AI systems provide real-time feedback and progress reports, allowing patients to stay informed about their treatment. This level of transparency enhances trust and engagement, as patients can see how their teeth are moving and what the expected outcomes are. The ability to monitor progress remotely also adds convenience, particularly for busy individuals who may struggle to attend frequent orthodontic appointments [10,62]. Patients also benefit from visualizing the entire treatment process through AI-generated 3D models, which show the expected final result before treatment even begins. This not only helps patients set realistic expectations but also increases their motivation and compliance with the treatment plan, as they can see the benefits of adhering to their aligner or brace schedules [77].

### 4.6. Technologies, Tools, and Workflow

The integration of Artificial Intelligence (AI) into orthodontic practice requires a combination of digital tools, specialized software, and advanced imaging technologies. For practitioners looking to streamline AI-powered treatment, a structured workflow incorporating AI-based diagnostics, treatment planning, and real-time monitoring is essential [44]. The first step in this digital orthodontic workflow is data acquisition, which involves high-resolution imaging techniques such as Cone Beam Computed Tomography (CBCT), intraoral scanners, and digital radiographs. These imaging systems provide AI-driven software with precise anatomical data, ensuring highly accurate diagnosis and treatment planning. AI-powered computer vision algorithms then analyze these images to detect malocclusions, tooth angulation, and jaw discrepancies, offering a level of diagnostic precision that surpasses traditional methods [40].

Once data is collected, orthodontists rely on AI-based treatment planning software, such as Align Technology’s ClinCheck (used in Invisalign), Dental Monitoring, OrthoAnalyzer, and 3 Shape Ortho System, to generate predictive models of tooth movement. These AI-powered platforms simulate multiple treatment scenarios, allowing practitioners to select the most efficient plan tailored to the patient’s specific needs [35]. AI optimizes aligner sequencing by predicting how each tooth will shift over time, reducing the number of refinements needed during treatment. Additionally, 3D printing technology is increasingly being used in conjunction with AI-driven design tools to fabricate custom aligners, retainers, and orthodontic appliances in-house, significantly cutting production time and improving patient convenience [78].

During treatment, real-time monitoring tools ensure accurate progress tracking with minimal in-office visits. AI-powered remote monitoring systems, such as Dental Monitoring and Grin Remote Monitoring, use smartphone-based intraoral scanning to assess tooth movement continuously. Patients can upload images using a mobile application, which AI software analyzes for deviations from the predicted movement pattern [31]. If any inconsistencies arise, the system alerts the orthodontist, who can then modify the treatment plan without requiring unnecessary clinic visits. AI-integrated robotic orthodontics, an emerging field, is also being explored for precise bracket placement and wire bending, reducing manual errors and enhancing treatment efficiency [40].

For orthodontists adopting AI-driven workflows, software interoperability and seamless integration with existing Electronic Health Records (EHRs) are critical. AI-powered platforms must be compatible with industry-standard digital orthodontic software such as Dolphin Imaging, OrthoCAD, and SureSmile, allowing for easy data sharing and efficient workflow management [40,79]. Additionally, cloud-based AI solutions enable practitioners to access treatment plans remotely, collaborate with other specialists, and improve case documentation. Implementing AI-driven orthodontics requires an investment in digital infrastructure, but its long-term benefits—precision, efficiency, and enhanced patient experience—make it a transformative tool in modern orthodontic care.

By leveraging these technologies, orthodontists can transition from conventional treatment planning to a fully AI-optimized approach, improving predictability while reducing treatment time and patient discomfort. The integration of AI in orthodontics is not merely an enhancement but a paradigm shift, streamlining workflows, improving decision-making, and setting new standards for personalized orthodontic care [80].

## 5. The Role of the Orthodontist in AI-Driven Treatment and Its Impact on Clinical Practice

The increasing use of AI in orthodontics does not replace the expertise of orthodontists but rather serves as a tool to enhance decision-making and streamline treatment planning. AI-powered tools can assist in diagnostics, automate treatment simulations, and offer remote monitoring; however, the role of an orthodontist remains essential, particularly in complex cases that require human expertise and critical thinking. The nuanced understanding of facial aesthetics, patient preferences, and individualized biomechanical considerations cannot be fully replicated by AI. Orthodontists must oversee AI-generated treatment plans to ensure accuracy, feasibility, and patient-specific modifications. Emphasizing this in clinical practice prevents any misconception that AI can function independently of trained specialists. AI should be viewed as an adjunct that improves efficiency while maintaining the orthodontist’s central role in patient care [42].

### 5.1. AI as a Tool, Not a Replacement: The Learning Curve and Time Considerations

AI streamlining treatment planning does not eliminate the need for orthodontic expertise; rather, it changes how specialists interact with data, requiring a learning curve to integrate AI-driven tools effectively [40]. Traditional orthodontic treatment planning can take hours or even multiple appointments, depending on case complexity, as it requires manual assessment of imaging, diagnosis, and iterative planning. AI-powered systems can significantly reduce this time by rapidly processing patient scans and generating predictive models, potentially cutting initial planning time in half. However, orthodontists must still validate AI-generated plans, interpret its recommendations, and make necessary refinements, which introduces a new time factor in the clinical workflow. While AI reduces the burden of repetitive tasks, it does not eliminate the orthodontist’s role in analyzing results and customizing treatments based on patient needs. Understanding the optimal balance between AI automation and human oversight will be key to ensuring both time efficiency and high-quality patient outcomes [15].

### 5.2. The Impact of AI on Patient Trust, Engagement, and Personalized Care

The adoption of AI in orthodontics raises concerns about how it may affect the human connection between orthodontists and patients. AI-powered remote monitoring and automated treatment planning have the potential to reduce in-person consultations, but the trust and comfort patients feel when interacting directly with their orthodontist remain crucial components of care. Patients may hesitate to rely solely on AI-generated recommendations, fearing a lack of personalized attention or the possibility of errors that only a human specialist can detect [10]. While AI enhances efficiency, it must not replace the direct patient–orthodontist relationship, which plays a key role in treatment adherence and overall satisfaction. Additionally, overuse of AI could lead to a decline in clinical intuition, as orthodontists may become overly reliant on AI-generated models rather than exercising independent judgment. To mitigate this, AI should be integrated in a way that supports rather than replaces the human element of orthodontic care. Establishing clear communication with patients about AI’s role, ensuring orthodontist oversight in decision-making, and maintaining regular in-person interactions will help preserve patient trust and engagement in the era of AI-driven orthodontics [15].

## 6. Future Prospects of AI in Orthodontics

**Figure 3:**
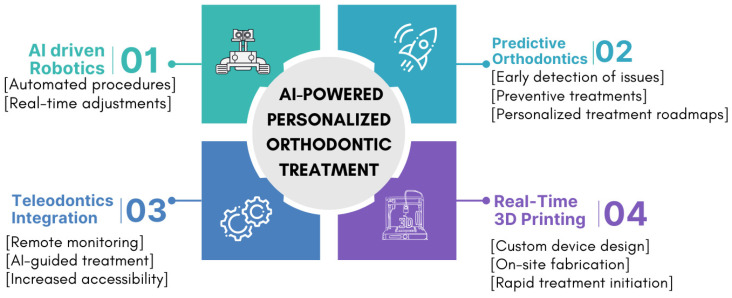
Future prospects of AI in orthodontics.

As AI continues to evolve, the future of AI-powered orthodontics is full of exciting possibilities that have the potential to revolutionize the field. These developments could drastically improve the precision, efficiency, and accessibility of orthodontic care, enhancing both patient outcomes and the practice of orthodontists. Figure 3 illustrates the main areas of future development for AI in orthodontics. This flow chart illustrates the main areas of future development for AI in orthodontics, including AI-driven robotics, predictive orthodontics, real-time 3D printing, and integration with tele-orthodontics, along with key aspects of each area.

### 6.1. AI-Driven Robotics in Orthodontic Procedures

One of the most exciting developments on the horizon is the integration of AI with robotics in orthodontics. Robotics is already making inroads in other areas of dentistry, such as implant placement, but the combination of AI’s predictive and analytical capabilities with robotic precision could lead to the automation of certain orthodontic procedures. AI-driven robots could perform tasks such as bracket placement, archwire adjustments, or even dental impressions with extreme precision, reducing human error and increasing efficiency [81]. By utilizing AI to guide robots in real-time, orthodontic procedures could become more standardized and consistent, leading to improved outcomes. Robots equipped with AI could also monitor the patient’s progress throughout the procedure and make adjustments autonomously based on real-time data, further enhancing precision and reducing treatment times [82]. This would not only elevate the quality of care but also make orthodontic procedures faster and more accessible to a larger patient population.

### 6.2. AI in Predictive Orthodontics

AI’s predictive capabilities are rapidly advancing, and the future may see AI being used to predict orthodontic needs much earlier in a patient’s life. By analyzing childhood dental records, 3D scans, genetic information, and other medical data, AI could identify potential misalignments or malocclusions before they fully develop [17]. This early detection could allow for preventive treatments during the formative years, potentially reducing or even eliminating the need for more extensive orthodontic interventions, such as braces or aligners, later in life. In predictive orthodontics, AI could provide orthodontists with a clear roadmap for guiding dental and jaw development, allowing them to intervene early and make small, non-invasive adjustments that would prevent more severe orthodontic issues in the future. This would not only improve long-term oral health outcomes but also reduce the overall cost and time involved in orthodontic care [1,34].

### 6.3. Real-Time 3D Printing of Orthodontic Devices

Another promising future application of AI in orthodontics is the combination of AI with 3D printing technology to create custom orthodontic devices in real-time. Currently, the fabrication of custom braces and aligners requires a certain lead time due to the design and manufacturing process. However, AI could streamline this process by rapidly analyzing patient data and generating custom designs, which could then be 3D printed on-site or even at a patient’s home [11,26]. For example, a patient could receive a 3D scan of their teeth during their initial consultation, and within minutes, an AI-driven system could generate a treatment plan and produce the first set of custom aligners or braces using a 3D printer. This real-time fabrication would eliminate the need for long wait times between consultations and the start of treatment, greatly improving patient satisfaction and the efficiency of orthodontic care. It could also enable more frequent adjustments, with AI systems creating new aligners or braces as the treatment progresses [11,52].

### 6.4. Integration with Tele-Orthodontics

Tele-orthodontics is already gaining traction, but its future integration with AI-powered systems could make orthodontic care more accessible than ever before. Remote monitoring tools powered by AI are already allowing patients to submit images of their teeth and receive feedback from orthodontists without needing to visit the clinic [83]. In the future, this could be expanded into a fully remote orthodontic experience, where patients can complete their entire treatment plan from home. AI could analyze patient data and guide the treatment process autonomously, only requiring human intervention in complex cases [84]. Orthodontists could monitor multiple patients simultaneously from a central location, offering consultations, reviewing progress, and adjusting treatment plans remotely. This approach could be especially beneficial in underserved areas, where access to orthodontic care is limited due to geographical or socioeconomic barriers [62,85]. Tele-orthodontics would also reduce the need for patients to travel for routine appointments, making orthodontic care more convenient and reducing the burden on clinics [86]. As AI-powered tools continue to evolve, they could enable a seamless integration between remote care and in-office visits, creating a hybrid model of orthodontic treatment that maximizes convenience and efficiency.

## 7. Ethical Considerations, Data Privacy, and Limitations of AI in Orthodontics

The integration of Artificial Intelligence (AI) in orthodontics presents significant advancements in precision, efficiency, and personalized care. However, as with any AI-driven healthcare solution, it raises critical ethical considerations, data privacy concerns, and inherent limitations that must be acknowledged and addressed. While AI has demonstrated great potential, issues related to biased datasets, algorithmic errors, overreliance on AI outputs, and the protection of patient data remain central challenges that require careful oversight [87,88].

### 7.1. Ethical Considerations in AI-Driven Orthodontics

AI-driven orthodontic systems rely on vast amounts of patient data, including 3D dental scans, X-rays, intraoral images, and medical histories. The collection, storage, and processing of this data pose significant privacy and security concerns [89]. One of the biggest risks is data breaches, where unauthorized access to sensitive patient information could compromise confidentiality [90]. Since AI models require large datasets for training and improvement, orthodontic AI companies must implement stringent data protection measures.

To safeguard patient data, AI-driven orthodontic systems must comply with international data privacy regulations such as the Health Insurance Portability and Accountability Act (HIPAA) in the United States and the General Data Protection Regulation (GDPR) in Europe [91]. These laws mandate that patient data be encrypted, securely stored, and accessible only to authorized personnel. Additionally, anonymization techniques can be used to strip personally identifiable information (PII) from datasets before they are used in AI training, ensuring that patient confidentiality is maintained.

Another key concern is data ownership. When AI companies collect large datasets for machine learning, questions arise about who owns the data and how it is used. Patients and practitioners must have clarity on whether data shared with AI software providers is stored locally, in the cloud, or shared with third parties [92]. Ethical AI implementation requires clear data governance policies, allowing patients to opt-in or opt-out of data-sharing agreements and ensuring that their information is used strictly for improving healthcare outcomes rather than for commercial exploitation.

### 7.2. Data Privacy and Security in AI-Orthodontic Applications

AI-driven orthodontic systems rely on vast amounts of patient data, including 3D dental scans, X-rays, intraoral images, and medical histories. The collection, storage, and processing of this data pose significant privacy and security concerns. One of the biggest risks is data breaches, where unauthorized access to sensitive patient information could compromise confidentiality. Since AI models require large datasets for training and improvement, orthodontic AI companies must implement stringent data protection measures.

To safeguard patient data, AI-driven orthodontic systems must comply with international data privacy regulations such as the Health Insurance Portability and Accountability Act (HIPAA) in the United States and the General Data Protection Regulation (GDPR) in Europe [91]. These laws mandate that patient data be encrypted, securely stored, and accessible only to authorized personnel. Additionally, anonymization techniques can be used to strip personally identifiable information (PII) from datasets before they are used in AI training, ensuring that patient confidentiality is maintained.

Another key concern is data ownership. When AI companies collect large datasets for machine learning, questions arise about who owns the data and how it is used. Patients and practitioners must have clarity on whether data shared with AI software providers is stored locally, in the cloud, or shared with third parties [92]. Ethical AI implementation requires clear data governance policies, allowing patients to opt-in or opt-out of data-sharing agreements and ensuring that their information is used strictly for improving healthcare outcomes rather than for commercial exploitation.

### 7.3. Limitations and Challenges of AI in Orthodontics

Despite its many benefits, AI-based orthodontic tools are not without limitations. Potential errors in automated diagnosis are a major challenge, as AI systems depend on the quality and comprehensiveness of their training data [87]. If an AI model has been trained on a dataset that lacks certain dental conditions, it may fail to recognize or accurately diagnose rare malocclusions, leading to incorrect or suboptimal treatment recommendations. This highlights the need for continuous model improvement, where AI systems are regularly updated with diverse and high-quality datasets to enhance diagnostic accuracy.

Another concern is the variability in AI performance across different patient demographics. Since AI models are trained on existing patient data, they may not perform equally well for all ethnicities, age groups, or unique dental cases. For instance, studies have shown that AI-based medical imaging tools can sometimes be less accurate for patients from underrepresented groups [93]. To address this, AI training datasets must be diverse and inclusive, ensuring that AI models perform reliably across all patient populations.

Additionally, over-reliance on AI without orthodontist verification can lead to significant risks. AI-generated treatment plans should always be reviewed by a qualified orthodontist to validate their feasibility. Blindly following AI recommendations without critical assessment could result in suboptimal patient care, particularly if an AI model fails to consider certain biomechanical or patient-specific factors [94]. AI should function as a complementary tool, providing recommendations that orthodontists can refine based on their clinical expertise.

Another challenge in AI-driven orthodontics is the high cost of implementation. AI-powered systems, including advanced imaging tools, treatment planning software, and cloud-based AI services, require substantial financial investment [80]. Many orthodontic practices, particularly smaller clinics, may find the cost of integrating AI prohibitive. To promote widespread adoption, AI technologies must become more affordable and accessible, with flexible pricing models that allow clinics to adopt AI tools without significant financial strain.

## 8. Conclusions

AI-powered personalized orthodontic treatment planning represents a significant advancement in dental care, offering unprecedented precision, efficiency, and customization. By leveraging AI’s sophisticated predictive capabilities, orthodontists are able to develop highly individualized treatment plans that not only improve patient outcomes but also significantly reduce treatment times. AI’s ability to predict tooth movement, optimize aligner and brace design, and continuously monitor treatment progress allows for a more effective and streamlined approach to orthodontic care. Despite the many benefits, challenges such as data security, algorithmic bias, and the need for widespread adoption remain. These obstacles must be addressed to ensure that AI technologies are applied ethically and equitably across diverse patient populations. As AI continues to evolve and integrate with other advanced technologies like robotics and 3D printing, the future of orthodontics will likely become more accessible, cost-effective, and patient-centered. The ongoing development of AI holds the promise of transforming not only the patient experience but also the overall landscape of orthodontic care, leading to better dental health outcomes on a global scale.

## Figures and Tables

**Table 1:** Benefits of AI-powered personalized orthodontic treatment across different patient categories.

Patient Category	Common Orthodontic Needs	How AI Enhances Treatment	Key Benefits	Challenges and Considerations
Children (6–12 years) [36,40]	Early intervention for developing malocclusions, space maintainers, monitoring growth patterns.	AI assists in early diagnosis through predictive modeling, detects jaw discrepancies, and automates cephalometric analysis.	Early treatment reduces need for extensive future interventions, improves facial growth, enhances treatment efficiency.	Requires long-term monitoring; AI predictions for growing patients must account for skeletal changes.
Teenagers (13–18 years) [10,40]	Comprehensive orthodontic treatment (braces, aligners), correction of malocclusions, compliance monitoring.	AI optimizes bracket/aligner placement, predicts treatment progress, and improves compliance tracking with remote monitoring.	Reduces treatment time, increases adherence through AI-driven reminders, improves aesthetics and confidence.	Teen compliance varies; AI-based remote tracking depends on patient engagement.
Adults (19–40 years) [37,41]	Aesthetic-focused orthodontics, mild-to-moderate malocclusions, clear aligners, relapse correction.	AI customizes aligners for precise tooth movement, shortens treatment time, integrates with digital smile design.	Enables minimally invasive treatment, enhances efficiency, provides virtual treatment simulations for better patient decision-making.	AI must account for periodontal health and bone density variations in adults.
Older Adults (40+ years) [42,43]	Orthodontic treatment with periodontal considerations, pre-prosthetic orthodontics, jaw alignment issues.	AI assesses bone loss, suggests adaptive treatment plans, and monitors periodontal risk factors in treatment planning.	Enhances orthodontic feasibility in complex cases, prevents worsening of periodontal issues, facilitates interdisciplinary care.	AI’s accuracy is influenced by dental history, bone density variability, and coexisting oral conditions.
Patients with Severe Skeletal Malocclusions [36,44]	Skeletal discrepancies requiring orthognathic surgery, severe overbites/underbites, facial asymmetry.	AI models simulate treatment outcomes, predict surgical-orthodontic needs, and assist in virtual surgical planning.	Improves surgical precision, allows better pre-treatment planning, provides visual predictions for patient understanding.	AI cannot replace surgeon expertise; accuracy depends on detailed 3D imaging integration.
Patients with Mild to Moderate Malocclusions [40,45]	Crowding, spacing, mild bite issues suitable for clear aligners or short-term braces.	AI-driven aligner design optimizes force application, predicts faster treatment timelines, and automates case selection.	Reduces treatment duration, enhances patient comfort, minimizes need for refinements.	AI performance varies depending on malocclusion complexity and patient compliance.
Patients with Orthodontic Relapse [10,46]	Post-treatment shifting of teeth, need for retainers, minor corrective aligner treatments.	AI detects minor shifts in tooth position, recommends retainer adjustments, and optimizes minor corrective movements.	Prevents further relapse, improves long-term stability, reduces need for extensive re-treatment.	Requires accurate post-treatment monitoring and patient adherence to retainer use.

**Table 2:** Machine learning models and their applications in AI-driven orthodontic treatment.

AI Application in Orthodontics	Machine Learning Model Used	Function and Role in Orthodontics	Advantages	Limitations
Cephalometric Landmark Detection and Tracing [10,40]	Convolutional Neural Networks (CNNs), Deep Learning Algorithms	Automates the identification of anatomical landmarks on cephalometric X-rays, aiding in diagnosis and treatment planning.	Reduces manual tracing time, enhances accuracy, minimizes intra-operator variability.	AI may misidentify landmarks in cases of poor image quality or anatomical variation, requiring orthodontist intervention.
Tooth and Jaw Segmentation in 3D Imaging [69]	U-Net, Region-Based CNN (R-CNN)	AI-driven segmentation of teeth and jawbones in CBCT, intraoral scans, and panoramic radiographs.	Provides detailed 3D models for accurate diagnosis and treatment planning, enhances aligner or bracket positioning.	AI accuracy depends on the quality of training data; complex cases (e.g., supernumerary teeth) may pose challenges.
AI-Powered Tooth Movement Prediction [35]	Recurrent Neural Networks (RNNs), Long Short-Term Memory (LSTM) Networks	Predicts how teeth will move under various orthodontic forces based on patient-specific biomechanical data.	Enables personalized treatment planning, reduces the need for mid-treatment refinements, optimizes force application.	Predictions may not always account for biological variability in tooth movement, requiring real-world validation.
Automated Orthodontic Treatment Planning [35,40]	Support Vector Machines (SVM), Gradient Boosting Algorithms (e.g., XGBoost)	AI-assisted optimization of treatment plans for braces and clear aligners based on patient-specific factors.	Increases efficiency in case analysis, allows orthodontists to evaluate multiple treatment simulations quickly.	AI-generated plans require orthodontist oversight; AI may not fully account for patient preferences or lifestyle factors.
Custom Aligner Fabrication [26,52]	Generative Adversarial Networks (GANs), Reinforcement Learning	AI optimizes the design of clear aligners based on 3D scans, ensuring precise force application for tooth movement.	Improves accuracy in aligner staging, reduces production costs, shortens overall treatment time.	Requires integration with 3D printing technology; miscalculations in force application can lead to mid-treatment refinements.
AI-Driven Bracket Positioning for Fixed Appliances [60]	CNN-Based Object Detection (YOLO, Faster R-CNN)	AI suggests optimal bracket placement on teeth, improving efficiency in fixed orthodontic treatments.	Enhances consistency and precision, reduces errors in manual placement.	May require manual adjustments in complex cases; AI models need continual refinement.
AI-Based Caries and Periodontal Disease Detection [10,35]	Deep Learning CNNs, Transfer Learning Models	AI detects early signs of caries, periodontal disease, and bone loss from intraoral scans and X-rays.	Enables early intervention, reducing long-term complications and improving oral health.	AI may misinterpret artifacts in imaging; false positives or negatives require human validation.
AI-Assisted Orthodontic Remote Monitoring [10,35]	Vision Transformers (ViTs), Deep CNNs	AI analyzes patient-submitted images and videos to track treatment progress remotely.	Reduces in-person visits, increases treatment adherence through real-time monitoring.	Image quality and patient compliance in capturing clear intraoral images may impact AI accuracy.
AI in 3D Printing for Orthodontics [26,52]	Generative Design Algorithms, Evolutionary AI	AI-driven customization of orthodontic appliances, including aligners, retainers, and splints, using 3D printing technology.	Reduces appliance production time, enables personalized treatment.	Integration with AI-driven design is still evolving, requiring orthodontist supervision.
AI-Driven Robotics in Orthodontics [71]	Reinforcement Learning, Motion Planning Algorithms	AI-assisted robotic systems for wire bending, bracket placement, and appliance customization.	Increases precision, reduces manual effort, improves consistency in orthodontic procedures.	High implementation costs; robotics in orthodontics is still in developmental stages.
